# The Barriers to Seeking Mental Health Services at King Saud Bin Abdulaziz University for Health Sciences

**DOI:** 10.7759/cureus.45321

**Published:** 2023-09-15

**Authors:** Meshal Alaqeel, Faris A Alkhudairy, Abdullah S Basuliman, Abdullah M Alsubaie, Faisal N Alqahtani, Hanan A Almkainzi

**Affiliations:** 1 Psychiatry, King Abdulaziz Medical City, Riyadh, SAU; 2 Medicine, King Abdulaziz Medical City, Riyadh, SAU; 3 Medicine, King Saud Bin Abdulaziz University for Health Sciences, Riyadh, SAU; 4 Internal Medicine, King Saud Bin Abdulaziz University for Health Sciences, Riyadh, SAU

**Keywords:** students health, mental health, saudi arabia, anxiety, depression

## Abstract

Background: The mental health of college students, especially medical students, is a major issue worldwide. Depression and anxiety are among the top causes of death among people aged 15-29 years old. Mental health disorders, especially mood disorders such as depression and anxiety, are common among university students. Psychological problems can negatively impact academic performance and life satisfaction. The earlier mental illness is detected, the better the treatment and outcome. The aim of this study is to determine the barriers among students to seeking mental health services.

Methods: This cross-sectional questionnaire-based study was carried out at King Saud bin Abdulaziz University for Health Sciences (KSAU-HS), Riyadh, Saudi Arabia The cross-sectional questionnaire-based study involved 434 students: 72.1% men and 27.9% women. This study had a response rate of 28.5% and responses were gathered in May 2023.

Results: The top three barriers identified in this study were “Feeling that my problems are not important,” “Concern that no one will understand my problems,” and “Difficulty with access to care.” The majority of students reported feeling “I feel reluctance a little” to use mental health services; 31.4% chose “I need it a little” when asked whether they needed to use mental health services. Furthermore, 34.8% of students reported having mild anxiety, and 34.1% reported having mild depression.

Conclusion: Mental illness is a serious issue, which is why medical students should be aware of it to improve their quality of life and reduce the stress and obstacles they face. Medical schools should address awareness of mental illness and how to approach a clinic. This is important for students to succeed and overcome the psychological difficulties that might affect academic performance. In addition, recognizing the barriers will help achieve better outcomes in seeking help and utilizing existing services.

## Introduction

Youth psychological well-being is important all over the world [1]. According to the World Health Organization, young people’s mental health issues are a growing concern. Depression and suicide are the second and third leading causes of death among people aged 15-29 years [2]. Mental health is defined as a person’s ability to remain positive in the face of adversity and stress. Furthermore, it plays a significant role in a person’s overall well-being for them to function normally and be able to work, learn, and contribute to society [3]. Anxiety and depression share the same presentations, such as insomnia, fatigue, irritability, and muscle tension, as stated by the American Psychological Association. Anxiety is present irrespective of a tangible external factor, unlike stress, which tends to be inflicted by a factor and persist for a short period [4].

Approximately 12%-50% of university students globally meet at least one diagnostic criterion for one or more mental health disorders [5]. In a large cross-sectional study in France, students were given a brief version of the Composite International Diagnostic Interview. The results showed that 8.5% had depressive episodes and 21.6% were diagnosed with anxiety disorders [6]. Another study carried out at Punjab University showed that 59.2% of students had depression, 86.5% had anxiety, and 52.7% had stress. Moreover, the study found a proportional relationship between the prevalence of these conditions and age. Women had a higher prevalence than men [7]. The prevalence of depressive symptoms among Chinese college students was approximately 24% [8].

In a study conducted in approximately 26 countries in Latin America, the Caribbean, Africa, and Asia, 24% of participants had moderate depressive symptoms, and 12.8% had severe depressive symptoms [9]. Another study showed that students with depression, anxiety, and stress tend to have low life satisfaction [10]. Mental illness can affect academic performance as well as life satisfaction levels, which may lead to low quality of life [11]. Only a small percentage of students recognize the symptoms of their mental health problems and seek professional health support and treatment [12]. Earlier intervention for mental illnesses results in improved outcomes and effective management [13]. The aim of this study is to determine the barriers to seeking mental health services among King Saud Bin Abdulaziz University for Health Sciences (KSAU-HS) students.

This article was previously posted to the Research Square preprint server on August 16, 2023.

## Materials and methods

Study settings and sample size: This cross-sectional questionnaire-based study was carried out at KSAU-HS, Riyadh, Saudi Arabia. It has a student wellness center at the College of Medicine that provides all services needed to psychologically support students throughout their academic life.

A convenience sampling technique was used. All current students enrolled in the KSAU-HS College of Medicine from the fourth, fifth, and sixth years were included in the study.

Out of 1,524 medical students, 434 (28.5%) were enrolled. The questionnaire was created using Google Forms, and students were invited to participate via email. All responses were gathered in May 2023. Approval for the study was obtained from the King Abdullah International Medical Research Center, and consent was obtained from students before participating.

Study measures: The survey included demographic items (gender and academic year), the Patient Health Questionnaire (PQ-9), the General Anxiety Disorder Questionnaire (GAD-7), and the Barriers to Mental Health Help Questionnaire (BMHH).

The PHQ-9 is a valid and reliable assessment instrument incorporating the DSM-IV [14]. It uses nine questions, with a total score ranging from 0 (minimal depression) to 27 (severe depression), to assess for depressive disorders.

The GAD-7 includes seven questions to assess anxiety disorders, with a total score ranging from 0 (minimal anxiety) to 21 (severe anxiety). The GAD-7 has been shown to have both good reliability and validity [15].

**Figure 1:**
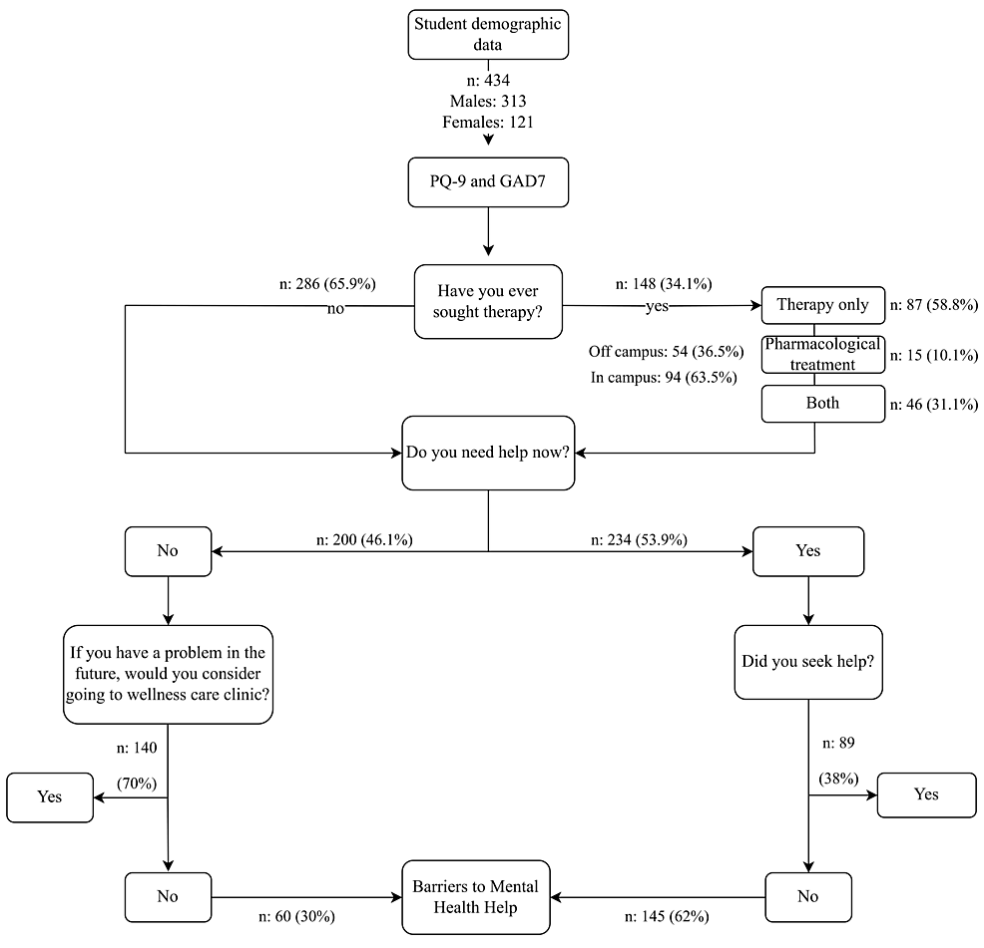
The students' process in answering the questionnaire.

The BMHH contains nine causes that prevent students from seeking mental health services; participants can choose up to three. The instrument items are “Feeling that my problems are not important,” “using services will mean that I am weak,” “difficulty with access to care,” “lack of confidentiality,” “concern that no one will be able to understand my problems,” “stigma of mental health care,” “fear of unwanted intervention,” “feeling that using services will mean that I’m weak,” “fear of documentation on academic record,” and “lack of availability of services.” This list of barriers was taken from a study by Givens et al. and modified to suit the sample size and culture [16]. Figure 1 describes the sample process for answering the questionnaire.

Data analysis: The data were analyzed using Statistical Product and Service Solutions (SPSS), version 26.0, software (IBM Inc., Armonk, NY, USA). Descriptive statistics (frequencies and percentages) were used to describe the categorical study and outcome variables. Pearson’s chi-square test was used to observe the association between the categorical variables and compare the distribution of proportions across two categorical variables. A p-value of ≤0.05 was used to report the statistical significance of the results.

## Results

Out of 434 participants, 72.1% were men, and 30% were sixth-year students. The rates of anxiety were 35% for mild, 19.2% for moderate, 13.8% for severe, and 32.3% for minimal. The rates of depression were 5.7% for no symptoms, 16.1% for minimal, 32.3% for mild, 25.3% for moderate, 14.8% for moderately severe, and 6% for severe symptoms (Table 1).

**Table 1:** Prevalence of anxiety and depression levels.

Anxiety and depression levels	n (%)
Anxiety level	
Minimal	139 (32)
Mild	152 (35)
Moderate	83 (19.2)
Severe	60 (13.8)
Depression level	
None	24 (5.5)
Minimal	70 (16.1)
Mild	140 (32.3)
Moderate	110 (25.3)
Moderately severe	64 (14.8)
Severe	26 (6)

More than 60% of the students did not seek psychological therapy either on or off campus. Among the 148 (33.4%) who sought psychological therapy, 58.8% had only therapy treatment, and 31.3% had both therapy and pharmacological treatments. Only 70% of students said that they would consider seeking help if they have a problem in the future. Regarding reluctance to use mental health services, 29.8% did not feel reluctant at all, whereas 16.9% felt reluctant strongly or very strongly. Concerning the need for mental health services, only 22.2% of students reported needing them strongly or very strongly, and 25.7% reported not needing mental health services. Of 234 participants, only 38% sought help for their mental health problems, and 70% of 200 participants were willing to consider going to a wellness care clinic if they had a problem in the future (Table 2).

**Table 2:** Distribution of study variables related to barriers to seeking mental health services (n=434).

Variable	n (%)
Gender	
Male	313 (72.1)
Female	121 (27.9)
Year of study	
3rd	87 (20.1)
4th	103 (23.7)
5th	114 (26.2)
6th	130 (30)
Ever sought psychological therapy	
Yes, on campus	94 (21.5)
Yes, off-campus	54 (12.4)
No	286 (65.9)
Do you feel reluctant to use mental health services?	
I don’t feel reluctance at all	129 (29.8)
I rarely feel reluctance	64 (14.7)
I feel reluctance a little	167 (38.5)
I feel reluctance strongly	48 (11.1)
I feel reluctance very strongly	26 (5.9)
Do you think you need some mental health services?	
I don’t need it at all	112 (25.7)
I rarely need it	88 (20.2)
I need it a little	137 (31.4)
I need it strongly	61 (14.0)
I need it very strongly	36 (8.2)
Did you seek help? (n=234)	
Yes	89 (38)
No	145 (62)
If you have a problem in the future, would you consider going to a wellness care clinic? (n=200)	
Yes	140 (70)
Yes	60 (30)

The participants were asked to give multiple responses on the different types of barriers to seeking mental health services. Out of the 207 who responded, “feeling that my problems are not important” was a barrier for 44%, followed by “concern that no one will be able to understand my problem” for 37.2%, “difficulty with access to care” for 32.4%, and “lack of confidentiality” for 31.9%. The other five barriers to seeking mental health services were reported by 14%-22.2% of participants (Table 3).

**Table 3:** Distribution of barriers to seeking mental health services in relation to depression and anxiety. * multiple responses

Type of barrier*	Total (%)	Depression	Anxiety
Feeling that my problems are not important	91 (44)	91	90
Concern that no one will be able to understand my problems	77 (37.2)	77	77
Difficulty with access to care	67 (32.4)	67	66
Lack of confidentiality	66 (31.9)	66	66
Using services will mean that I am weak	46 (22.2)	46	45
Fear of documentation on academic record	42 (20.3)	42	41
Fear of unwanted intervention	38 (18.4)	38	37
Lack of availability of services	31 (15.0)	31	31
Stigma of mental health care	29 (14.0)	29	29

## Discussion

This study aimed to identify barriers preventing students from seeking mental health support. It identified four major barriers in the literature, which shed light on the barriers to seeking clinical psychology mental health support for medical students. The results showed that the most prominent reasons deterring the students from seeking psychological help were feeling that their problems were not important (44%), concern that no one would be able to understand their problems (37.2%), difficulty with access to care (32.4%), and lack of confidentiality (31.9%). As found by Givens et al., more than 30% of obstacles were related to the lack of confidentiality, concern that “no one will understand my problems,” and feeling that “my problems are not important” [16].

The first, second, and fifth barriers, “feeling the issue is not important,” “no one will understand my problems,” and “using the services means the person is weak,” respectively, can be overcome by increasing awareness regarding each issue the person faces, shedding light on the fact that, no matter what a person goes through, it still matters and they should seek help. The third barrier was difficulty in accessing care, such as inconvenience, lack of knowledge about available services, and transportation. Telehealth can overcome this barrier for people who cannot go to clinics by providing internet- or app-based sessions that can improve access to mental health treatments and have shown effectiveness of up to 47% [17]. In addition, conducting online sessions for half an hour helped reduce the severity of mental illness symptoms, as reported by Schleider et al. [18].

The barriers “lack of confidentiality” and “fear of documentation on academic records” ranked fourth and sixth, respectively. These barriers can be overcome by providing emotional and academic support and ensuring that everything reported to the student wellness clinic remains confidential. This can encourage students to seek therapy [19]. Fear of unwanted intervention ranked as the seventh barrier. Students should be educated that early intervention in mental illness is beneficial and leads to positive outcomes, as stated by Membride [13].

“Lack of availability of services” ranked as the eighth barrier. Saudi Arabia is a country that provides free health services, yet mental health professional numbers are low, according to a study by Al-Subaie et al. [20]. More mental health professionals are needed to help fill the gap in regard to the proportion of psychiatrists in Saudi Arabia to the global average, which will guarantee an increase in the mental health services provided in the country. Furthermore, this barrier shows that universities should begin promoting and increasing awareness regarding their mental health services and encourage students to use them [20]. The last barrier was “the stigma of mental health.” According to Henderson, stigma may increase the likelihood of treatment avoidance as well as the discontinuation of service use. Addressing public stigma in society may lead to decreased stigma among service users and facilitate help-seeking and engagement with mental health care [21].

According to a study by Baklola et al. [22], 70.8% of participants did not seek help due to multiple barriers. The first barrier, “wanting to solve the problem on my own,” was reported by 45.5% of participants. The second barrier, “disliking talking about feelings, emotions, or thoughts,” was reported by 48% of participants. The third barrier, “being unsure where to go to get professional care,” was reported by 47.1% of participants. These barriers are similar to those in the current study. This will aid in shedding light on such barriers to improve the quality of services and awareness regarding seeking mental help [22].

Alangari et al. [23] reported that the most frequent barrier was that people wanted to handle issues by themselves. The second barrier was thinking that their problem was not important, and the third barrier was that the available services were not effective. Alangari et al.’s findings are similar to this research paper in regard to “feeling that my problems are not important,” “concern that no one will be able to understand my problems,” “lack of availability of services,” and “difficulty of access to care.” In contrast, the stigma of mental healthcare was the least reported barrier, whereas, in Alangari et al.’s research paper, it was listed as the third attitudinal barrier. This difference may be due to increased awareness of mental health issues in the last five years [23].

These papers support the claim of existing barriers preventing students from seeking support for underlying mental illnesses. Foremost, these results signify how and why students refrain from seeking mental health support. Most of the barriers reported were not related to the therapy itself or past experiences, which can be facilitated to encourage students to pursue mental health support.

This paper helps identify the barriers for healthcare practitioners and other stakeholders to incorporate methods to ease the process for students’ well-being. For future questions regarding this field, we recommend increasing the sample size to include multiple health science colleges and premedical students. Furthermore, a qualitative study is better to go in-depth regarding those barriers and how to overcome them from a student’s perspective.

Limitations

The findings of this study must be seen in light of some limitations. Being an online rather than on-site survey has prolonged the data collection period and may have limited the number of participants. To improve this limitation in future papers, we recommend that the research question be asked on a grander scale across all health science colleges to obtain an accurate view of possible barriers across multiple fields of health studies and how these might vary geographically. Additionally, the on-site distribution of the survey could ensure an even more holistic sample to improve future findings.

## Conclusions

In conclusion, mental health problems are a well-documented issue in higher education. Recognizing and addressing mental health issues and the barriers to seeking professional help early are linked to favorable results and improved outcomes. Finding and implementing solutions to the barriers is only achievable after the identification of these barriers. By using validated questionnaires and established scales, this study found that more than 60% of participants did not seek professional help when needed, with varying barriers. The perceived barriers significantly differed by the state of underlying mental illness, gender, previous psychological therapy, reluctance to seek therapy, and perception of needing therapy.

The study also showed that female participants experienced more anxiety than their male counterparts, and the severity of anxiety correlated with the reluctance to seek mental health services and the perception of needing such services. Furthermore, female participants exhibited more severe depression than male participants, and the level of depression correlated with the reluctance to seek mental health services and the perception of needing mental health services.
